# Low Left Atrial Compliance Contributes to the Clinical Recurrence of Atrial Fibrillation after Catheter Ablation in Patients with Structurally and Functionally Normal Heart

**DOI:** 10.1371/journal.pone.0143853

**Published:** 2015-12-01

**Authors:** Junbeom Park, Pil-sung Yang, Tae-Hoon Kim, Jae-Sun Uhm, Joung-Youn Kim, Boyoung Joung, Moon-Hyoung Lee, Chun Hwang, Hui-Nam Pak

**Affiliations:** 1 Yonsei University Health System, Seoul, Republic of Korea; 2 Utah Valley Medical Center, Provo, Utah, United States of America; Universitätsklinikum des Saarlandes, GERMANY

## Abstract

Stiff left atrial (LA) syndrome was initially reported in post-cardiac surgery patients and known to be associated with low LA compliance. We investigated the physiological and clinical implications of LA compliance by estimating LA pulse pressure (LApp) among patients with atrial fibrillation (AF) and structurally and functionally normal heart. Among 1038 consecutive patients with LA pressure measurements before AF ablation, we included 334 patients with structurally and functionally normal heart (81.7% male, 54.1±10.6 years, 77.0% paroxysmal AF) after excluding those with hypertension, diabetes, and previous ablation or cardiac surgery. We measured LApp (peak-nadir LA pressure) at the beginning of the ablation procedure and compared the values with clinical parameters and the AF recurrence rate.AF patients with normal heart were younger and more frequently male and had paroxysmal AF, a lower body mass index, and a lower LApp compared to others (all p<0.05).Based on the median value, the low LA compliance group (LApp≥13mmHg) had a smaller LA volume index and lower LA voltage (all p<0.05) compared to the high LA compliance group. During a mean follow-up of 16.7±11.8 months, low LA compliance was independently associated with two fold-higher risk of clinical AF recurrence (HR:2.202; 95%CI:1.077–4.503; p = 0.031).Low LA compliance, as determined by an elevated LApp, was associated with a smaller LA volume index and lower LA voltage and independently associated with higher clinical recurrence after catheter ablation in AF patients with structurally and functionally normal heart.

## Introduction

Stiff left atrial (LA) syndrome, was initially reported by Pilote et al. in 1988, presenting as severe pulmonary hypertension 7 years after mitral valve surgery.[1]In their report, cardiac catheterization revealed a marked V wave without any mitral prosthetic valve dysfunction or regurgitation. Thus, stiff LA syndrome has generally been defined as pulmonary hypertension that develops long after cardiac surgery without any other cardiac cause.[1]However, this syndrome regained attention in patients who had undergone catheter ablation for atrial fibrillation (AF), especially after multiple ablation procedures.[2]Although there have been reports of more extensive ablation, resulting in better clinical outcomes in patients with persistent AF (PeAF),[3] more touches may reduce LA compliance. Consequently, pulmonary hypertension after catheter ablation is detected in 1.4% of patients without pulmonary vein (PV) stenosis. It is also reported to occur more in patients with previous LA scarring and small LA dimensions.[2]Recently, we reported that high LA pressures are associated with both advanced electroanatomical remodeling of LA and independent predictors for clinical recurrence of AF after catheter ablation.[4]However, it is unclear whether LA compliance has some clinical implication in patients who underwent radiofrequency catheter ablation (RFCA). Therefore, we hypothesized that the reduced LA compliance itself may contribute to the pathophysiology of AF, even though it is not as extreme as stiff LA syndrome. Considering that AF is a multifactorial disease associated with degenerative processes, such as aging, hemodynamic factors, and metabolic factors, we tested our hypothesis in AF patients without hypertension, diabetes, or associated cardiac disease, after excluding for other confounding factors. We also attempted to identify factors that contributed to LA compliance. Although there are several different indirect ways to estimate LA compliance,[5,6] we quantified LA compliance by directly measuring the LA pulse pressure (LApp) at the beginning of the procedure and assumed minimal change in LA volume.[5–7]

## Material and Methods

### Study population

The study protocol adhered to the Declaration of Helsinki and was approved by the Institutional Review Board of Yonsei University Health System. All patients provided written informed consent. From1038 consecutive patients with LA pressure measurements in the Yonsei AF Ablation Cohort (June 2009 to December 2013), we identified 334AF patients with structurally and functionally normal heart after excluding certain patients due to hemodynamic and metabolic factors. The study’s exclusion criteria were as follows: 1) permanent AF refractory to electrical cardioversion; 2) associated hypertension, diabetes, or congestive heart failure; 3) E/Em>15 suggesting left ventricular (LV) diastolic dysfunction; 4) AF with any valvular disease (including mild-degree disease); 5) structural heart disease; 6) coronary artery disease with luminal narrowing of more than 50% or history of percutaneous coronary intervention; 7) prior cardiac surgery or AF catheter ablation; and 8)unmeasurable LA pressure (LAP) during sinus rhythm due to frequent re-initiation of AF. The majority (76.6%) of the patients had paroxysmal AF (PAF), and 23.4% had PeAF. Three-dimensional (3D) spiral computerized tomography (CT) scans (64 Channel, Light Speed Volume CT, Philips, Brilliance 63, Netherlands) were performed to define the pulmonary vein (PV) anatomy. All antiarrhythmic drugs were discontinued for a period corresponding to at least five half-lives. Anticoagulation therapy was maintained prior to catheter ablation.

### Echocardiographic evaluation of the heart

**Fig 1:**
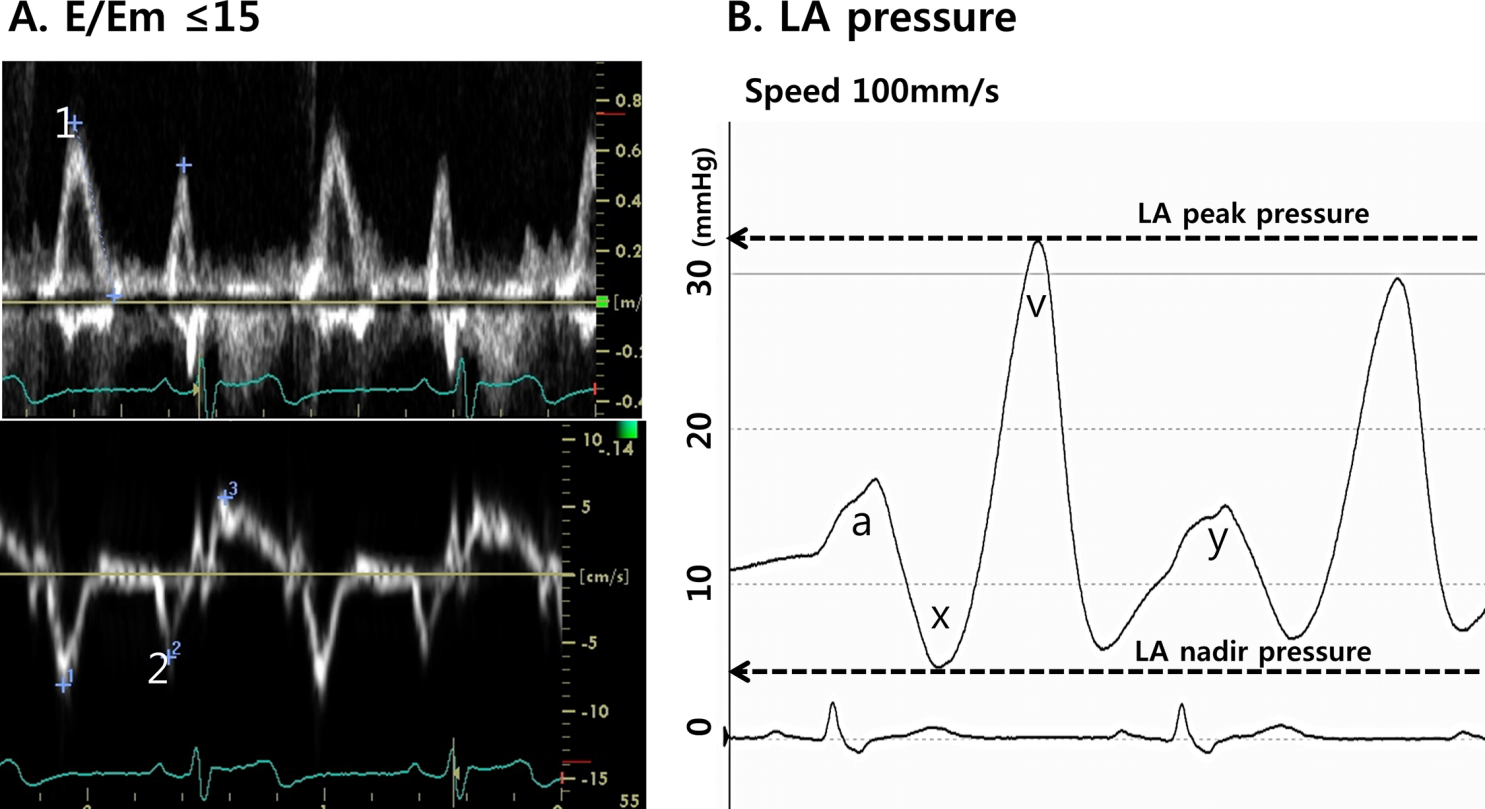
(A) Tissue Doppler images of the mitral annular septal area (peak diastolic velocity [Em]) and the flow velocity of mitral inflow were acquired. Patients with E/Em>15, suggesting left ventricular (LV) diastolic dysfunction, were excluded. (B) LAP_peak_ (v wave), LAP_nadir_ (x wave), and LAP_mean_ were measured during sinus rhythm, and LA pulse pressure (LApp) was calculated by subtracting LAP_nadir_ from LAP_peak_ (the difference between LAP_peak_ and LAP_nadir_).

All patients underwent transthoracic echocardiography prior to RFCA. Chamber size (LA volume index [LAVI], LA dimension, LV wall thickness, and LV mass index [LVMI]), transmitral flow velocity (E wave, A wave), and tissue Doppler images of the mitral annular septal area (peak diastolic velocity [Em] and peak systolic velocity [Sm]) were acquired according to the American Society of Echocardiography guidelines (Fig 1A).The index was calculated as divided by body surface area (BSA). Transesophageal echocardiography (TEE) was performed in order to exclude intracardiac thrombi and measure the emptying velocity of the LA appendage in all patients prior to ablation using the average derived from five consecutive beats.

### Left atrial pressure measurement

Intracardiac electrograms and hemodynamic measurements were recorded using the Prucka Cardio Lab^TM^ electrophysiology system (General Electric Medical Systems Inc., Milwaukee, WI, USA). A double transseptal puncture approach was used for catheter access to the LA. LAP was measured during both sinus rhythm and AF immediately after transseptal puncture using a 6-Fr pigtail catheter (A&A Medical Device Inc., Gyeonggi-do, Republic of Korea) that was inserted into the LA via a long sheath (Schwartz left 1, St. Jude Medical Inc., Minnetonka, MN, USA).[4] If the initial rhythm was AF, we measured LAP insinus rhythm after internal cardioversion (2–10J biphasic shocks, Lifepak12, Physiocontrol Ltd.). We then measured LAP after waiting at least 3 minutes until recovery from atrial stunning by cardioversion. If we failed to maintain sinus rhythm after three cardioversion attempts, we excluded the patient from the study. We analyzed LAP_peak_ (v wave), LAP_nadir_ (xwave), and LAP_mean_for 10 consecutive cycles in sinus rhythm, and the results were averaged.[6] LA pulse pressure (LApp) was calculated by subtracting LAP_nadir_ from LAP_peak_ (the difference between LAP_peak_ and LAP_nadir_, Fig 1B).Atrial compliance was calculated asΔV/ΔP.[5,6] Several previous studies indirectly measured LA volume using different echocardiographic techniques.[5,6]However, precise and instantaneous measurement of LA volume change and pressure is technically challenging due to the complexity of LA geometry. Therefore, we defined and quantified LA compliances through direct measurement of LApp and assumed a minimal change in LA volume based on the previous physiologic studies.[5–7]The representation of aortic compliance by aortic pulse pressure was based on the same rationale.[8]

### Electroanatomical mapping and LA CT measurement

A 3D electroanatomical map (NavX, St. Jude Medical Inc., Minnetonka, MN, USA) was generated using a circular PV mapping catheter (Lasso; Biosense-Webster Inc., Diamond Bar, CA, USA). NavX-system-generated 3D geometries of the LA and PVs were merged with the corresponding 3D spiral CT images. We generated LA voltage maps by obtaining contact bipolar electrograms from 350–500 points on the LA endocardium during atrial pacing at 500ms and calculated the mean LA voltage as previously described.[9]LA voltage was measured at a point of secure endocardial contact by experienced operators after circumferential PV isolation. Contact artifacts, noises, and isolated PV area were excluded in voltage analysis. A technician who was blinded to the clinical information, analyzed the color-coded CT-merged NavX voltage maps with customized software (Image Pro, Media Cybernetics, Inc., Rockville, MD, USA).[9,10] The 3D spiral CT images of the LA were analyzed on an imaging processing workstation (Aquarius, Terarecon Inc., Foster City, CA, USA). For the regional volumetric analyses, each LA image was subdivided according to embryological origin as follows: venous LA, anterior LA, and LA appendage.[9]

### Radiofrequency catheter ablation

An open irrigated 3.5-mm-tip deflectable catheter (Celsius, Johnson & Johnson Inc., Diamond Bar, CA, USA; Coolflex, St. Jude Medical Inc., Minnetonka, MN, USA; 30–35 W; 47°C) was used for RFCA. All patients initially underwent circumferential PV isolation and cavotricuspid isthmus block. For the patients with PeAF, we added a roof line, a posterior inferior line, and an anterior line to the standard lesion set. Additional ablations of the superior vena cava, non-PV foci, and complex fractionated electrograms were conducted at the operator’s discretion. The procedure ended when there was no immediate recurrence of AF after cardioversion with isoproterenol infusion (5–10μg/min). If there were mappable AF triggers or atrial premature beats, we carefully mapped and ablated these non-PV foci as much as possible.

### Post-ablation management and follow-up

All patients were followed, and antiarrhythmic drugs were discontinued after RFCA. Patients visited an outpatient clinic regularly at 1, 3, 6, and 12 months and then every 6 months or whenever symptoms occurred after RFCA. All patients underwent electrocardiography (ECG) at every visit and 24- or 48-hour Holter recording or event recording at 3, 6, and every 6 months, following the 2012 Heart Rhythm Society (HRS) / European Heart Rhythm Association (EHRA) / European Cardiac Arrhythmia Society (ECAS) Expert Consensus Statement guidelines. However, whenever patients reported palpitations, Holter monitor or event monitor recordings were obtained and evaluated for possible recurrence of arrhythmia. We defined recurrence of AF as any episode of AF or atrial tachycardia of at least 30 sec in duration. Any ECG documentation of AF recurrence after 3 months was diagnosed as clinical recurrence.

### Data analysis

Normally-distributed continuous variables are expressed as mean ± standard deviation (SD). We compared LAP during both sinus rhythm and AF using the degree of electroanatomical remodeling of the LA, echocardiographic parameters reflecting hemodynamic status, and clinical outcomes. Statistical significance of the comparisons was assessed using a student *t-*test and χ^2^ test. And uni- and multi-variate logistic regression analyses were used to analyze the association between clinical parameters and LApp. Variables selected for multivariate analysis were those with p-value<0.05 on univariate analysis or with having association with AF recurrence clinically. And if there was significant correlation between selected variables (R>0.5), only one variable was used to avoid multicolinearity for multivariate regression analysis. Log minus log (LML) graph for analyzing the proportionality assumption of LApp shows parallel pattern according to groups of LApp. This means that the effects of LApp are constant regardless of time (the proportional assumption of Cox regression analysis). The cut off value for LApp, which best differentiate recurrence and no recurrence, was determined by an algorithm of maximization of hazard ratio,[11] this cut off value is identical with median (LApp = 13mmHg). Kaplan-Meier and Cox regression analysis were used to analyze AF-free survival after catheter ablation. Variance inflation factors (VIF)≥10 were considered as indicating co-linearity and were excluded in multivariate linear regression. A *p*-value <0.05 was considered statistically significant.

## Results

### Clinical characteristics of AF with structurally and functionally normal heart

Among the 1038 patients, 334 patients (32.2%)were classified as having AF with structurally and functionally normal heart after excluding any structural heart disease, hypertension, and diabetes (Table 1).

**Table 1:** Clinical characteristics of patients.

	All	Normal heart AF (n = 334)	Others	*p-*value
	(n = 1038)		(n = 704)	
**Male (n,%)**	771 (74.3%)	273 (81.7%)	498 (70.7%)	**<0.001**
**Age (years)**	57.7±11.2	54.1±10.6	59.5±11.1	**<0.001**
**PAF (n,%)**	704 (67.8%)	256 (76.6%)	448 (63.6%)	**<0.001**
**BSA (m** ^**2**^ **)**	1.8±0.2	1.8±0.2	1.8±0.2	0.053
**BMI (kg/m** ^**2**^ **)**	24.9±3.1	24.6±2.7	25.0±3.2	**0.041**
**CHA2DS2VASc score**	1.5±1.4	0.6±1.0	2.0±1.4	**<0.001**
**Heart Failure (n,%)**	89 (8.6%)	0	89 (12.6%)	NA
**Hypertension (n,%)**	488 (47.0%)	0	488 (69.3%)	NA
**Age≥75 (n,%)**	50 (7.1%)	4 (1.2%)	46 (6.5%)	**<0.001**
**Diabetes (n,%)**	140 (13.5%)	0	140 (19.9%)	NA
**Stroke/TIA (%, n)**	125 (12.0%)	24 (7.2%)	101 (14.3%)	**<0.001**
**Associated structural heart disease** ^*^	261 (37.1%)	0	261 (37.1%)	
**Coronary artery disease (n,%)**	142 (20.2%)	0	142 (20.2%)	NA
**Valvular heart disease (n,%)**	75 (10.7%)	0	75 (10.7%)	NA
**HCMP (n,%)**	20 (2.8%)	0	20 (2.8%)	NA
**DCMP (n,%)**	17 (2.4%)	0	17 (2.4%)	NA
**Congenital heart disease (n,%)**	17 (2.4%)	0	17 (2.4%)	NA
**LApp (mmHg)**	15.3±7.6	14.0±6.1	16.0±8.3	**<0.001**

PAF, paroxysmal atrial fibrillation; BSA, body surface area; BMI, body mass index; DCM, dilated cardiomyopathy; HCMP, hypertrophic cardiomyopathy; TIA, transient ischemic attack; LApp, left atrial pulse pressure

*, There are some overlapping of multiple structural heart diseases in the same patients.

We intentionally excluded patients with diabetes to rule out metabolic factors affecting LA compliance; however, the results were consistent when the patients with diabetes (n = 21) were included in the normal heart AF group (S1 Table).Patients in the normal heart AF group were younger and more likely to be male and to have paroxysmal AF (all p<0.001). Additionally, patients in the normal heart AF group had a lower body mass index (BMI) than other AF patients, and LApp in the normal heart AF group was significantly lower than in other AF patients (all p<0.05; Table 1).

### Low LA volume index and low LA voltage in AF with reduced LA compliance

**Fig 2:**
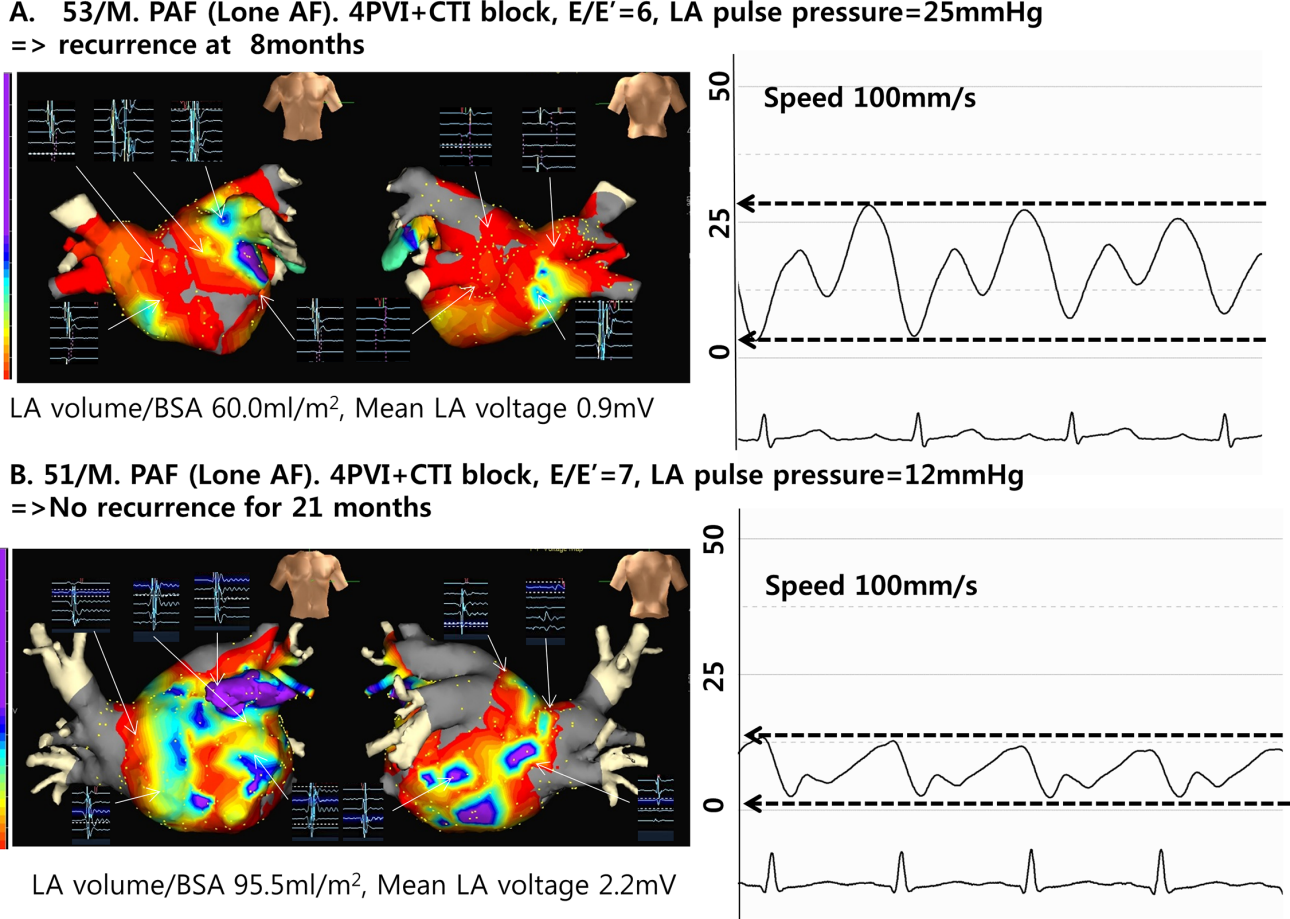
Typical examples of patients with low (A) or high (B) LA compliance. (Electroanatomical mapping was performed during high right atrial pacing 500ms.) Patients with low LA compliance (A) have relatively smaller LA volume and lower endocardial voltage than patients with high LA compliance and show poor clinical outcome of AF after RFCA.

For the 334normal heart AF patients, we defined low LA compliance as LApp≥13mmHg, based on the median value of LApp (Table 2). The cut off value by median is identical with value calculated by an algorithm of maximization of hazard ratio (LApp = 13mmHg).[11]Heart rate was not significantly different at the time of LAP measurement between the groups with high and low LApp. Both the LA volume index measured by 3D-CT and mean LA voltage were lower in patients with LApp≥13mmHg than in those with LApp<13mmHg (both p<0.05; Table 2, Fig 2).

**Table 2:** Baseline characteristics of patients according to the LA compliance.

	All	LApp (SR)≥13mmHg	LApp (SR)<13mmHg	*p-*value
	(n = 334)	(n = 186)	(n = 148)	
**Heart rate at LApp (bpm)**	59.4±9.5	59.2±10.1	59.6±8.6	0.731
**Male (%)**	81.7	80.1	83.8	0.389
**Age (years)**	54.1±10.6	54.3±10.5	53.8±10.8	0.645
**PAF (%)**	76.6	79	74.3	0.312
**BSA (m** ^**2**^ **)**	1.8±0.2	1.8±0.2	1.8±0.2	0.206
**BMI (kg/m** ^**2**^ **)**	24.6±2.7	24.3±2.7	24.9±2.7	0.053
**Stroke/TIA (%, n)**	7.2	8.1	6.1	0.487
**Echocardiogram**				
**LA dimension (mm)**	39.7±5.6	39.4±5.5	40.1±5.6	0.262
**LA volume index (ml/m** ^**2**^ **)**	30.8±9.6	30.2±9.1	31.5±10.2	0.230
**LV ejection fraction (%)**	63.9±6.4	63.7±6.0	64.3±6.9	0.406
**E/Em**	8.5±2.5	8.5±2.5	8.6±2.4	0.655
**LVMI (g/m** ^**2**^ **)**	88.3±18.3	87.1±17.7	90.0±19.0	0.228
**LAA emptying velocity (cm/s)**	56.0±22.8	56.8±21.4	55.2±24.1	0.631
**3D-CT**				
**LA volume/BSA (ml/m** ^**2**^ **)**	75.4±19.8	73.1±19.9	78.4±19.3	**0.035**
**Anterior LA/BSA (ml/m** ^**2**^ **)**	43.3±13.7	42.1±13.5	44.9±14.0	0.101
**LAA/BSA (ml/m** ^**2**^ **)**	6.7±3.2	6.5±3.0	7.0±3.5	0.229
**NavX Voltage Map (n = 123)**				
**Mean LA voltage (mV)**	1.3±0.7	1.2±0.6	1.4±0.8	**0.032**
**Mean LAA voltage (mV)**	2.5±1.6	2.3±1.5	2.8±1.7	**0.039**
**BNP (pmol/L)**	365.6±289.0	354.9±282.7	380.6±298.0	**0.455**
**Conduction Velocity (m/sec)**	0.5±0.4	0.5±0.4	0.6±0.4	0.293
**Ablation time (sec)**	4815.2±1456.3	4724.2±1492.6	4929.8±1406.0	0.203
**Procedure time (min)**	185.3±44.5	181.7±43.1	189.8±45.9	0.100
**Follow-up duration (months)**	16.7±11.8	15.9±12.5	17.8±10.9	0.862

SR, sinus rhythm; LApp, left atrial pulse pressure; PAF, paroxysmal atrial fibrillation; BSA, body surface area; BMI, body mass index; TIA, transient ischemic attack; LA, left atrium; LV, left ventricle; LVMI, left ventricular mass index; LAA, left atrial appendage; BNP, B-type natriuretic peptide.

In the multivariate logistic regression analysis, low LA voltage was independently associated with low LA compliance (LApp≥13mmHg) after adjusting age, gender, AF type, and LA volume (OR = 0.395;95% CI: 0.168–0.928;p = 0.033;Table 3).

**Table 3:** Multivariate regression analysis of factors related to low LA compliance (LApp ≥ 13mmHg).

	Univariate analysis			Multivariate analysis		
	OR	95% CI	*p*-value	OR	95% CI	*p*-value
Age	1.005	0.985–1.026	0.644	1.002	0.971–1.035	0.881
Male	0.779	0.442–1.373	0.388	0.697	0.283–1.717	0.433
Persistent AF	0.768	0.461–1.280	0.311	0.622	0.273–1.419	0.259
BSA (m^2^)	0.458	0.137–1.537	0.206			
BMI (kg/m^2^)	0.923	0.851–1.001	0.054			
LA dimension (Echo)	0.978	0.940–1.017	0.262			
LA volume index (Echo)	0.986	0.963–1.009	0.23			
LV ejection fraction	0.986	0.953–1.020	0.405			
E/Em	0.98	0.899–1.069	0.654			
LAA emptying velocity	1.003	0.991–1.016	0.629			
LA volume index (3D-CT)	0.986	0.974–0.999	**0.036**	0.983	0.966–1.001	0.069
Mean LA voltage (NavX)	0.666	0.460–0.964	**0.031**	0.395	0.168–0.928	**0.033**
Mean LAA voltage	0.841	0.715–0.991	**0.038**	1.048	0.749–1.467	0.784

BSA, body surface area; BMI, body mass index; LA, left atrium; LV, left ventricle; LAA, left atrial appendage. OR, odds ratio; CI, confidence interval

### Low LA compliance is independently associated with post-ablation AF recurrence

During a mean follow-up of16.7±11.8 months (range, 3 to 47 months), high LApp≥13mmHg (HR 2.202; 95% CI: 1.077–4.503; p = 0.031), early recurrence (HR 2.083; 95% CI: 1.042–4.166; p = 0.038) and mean LA voltage (HR 0.46; 95% CI: 0.249–0.850; p = 0.013) were strong predictors of clinical AF recurrence after RFCA, independent of age, gender, and AF type (Table 4).

**Table 4:** Multivariate Cox regression analysis of clinical AF recurrence after RFCA

	Univariate analysis			Multivariate analysis		
	HR	95% CI	*p*-value	HR	95% CI	*p*-value
**Age**	1.001	0.977–1.026	0.919	0.996	0.964–1.028	0.79
**Male**	1.153	0.583–2.280	0.682	1.798	0.662–4.886	0.25
**Persistent AF**	1.364	0.766–2.428	0.291	3.099	1.054–9.117	**0.04**
**BSA (m** ^**2**^ **)**	0.777	0.186–3.243	0.73			
**BMI (kg/m** ^**2**^ **)**	0.993	0.903–1.091	0.883			
**LA dimension (Echo)**	1.046	1.001–1.093	0.044	1.05	0.983–1.122	0.146
**LA volume index (Echo)**	1.022	0.998–1.047	0.068			
**LV ejection fraction**	1.002	0.964–1.041	0.935			
**E/Em**	0.981	0.882–1.091	0.718			
**LAA emptying velocity**	0.996	0.979–1.013	0.627			
**LA volume index (3D-CT)**	1.003	0.989–1.018	0.647			
**LAA volume index (3D-CT)**	1.031	0.939–1.132	0.523			
**Mean LA voltage**	0.528	0.310–0.899	0.019	0.46	0.249–0.850	**0.013**
**Mean LAA voltage**	0.827	0.663–1.031	0.091			
**Ablation time**	1	1.000–1.000	0.026	1	1.000–1.001	**0.034**
**Early recurrence**	2.935	1.749–4.927	<0.001	2.083	1.042–4.166	**0.038**
**LApp≥13mmHg**	1.773	1.022–3.075	0.042	2.202	1.077–4.503	**0.031**

BSA, body surface area; BMI, body mass index; LA, left atrium; LV, left ventricle; LAA, left atrial appendage; LApp, left atrial pulse pressure.

**Fig 3:**
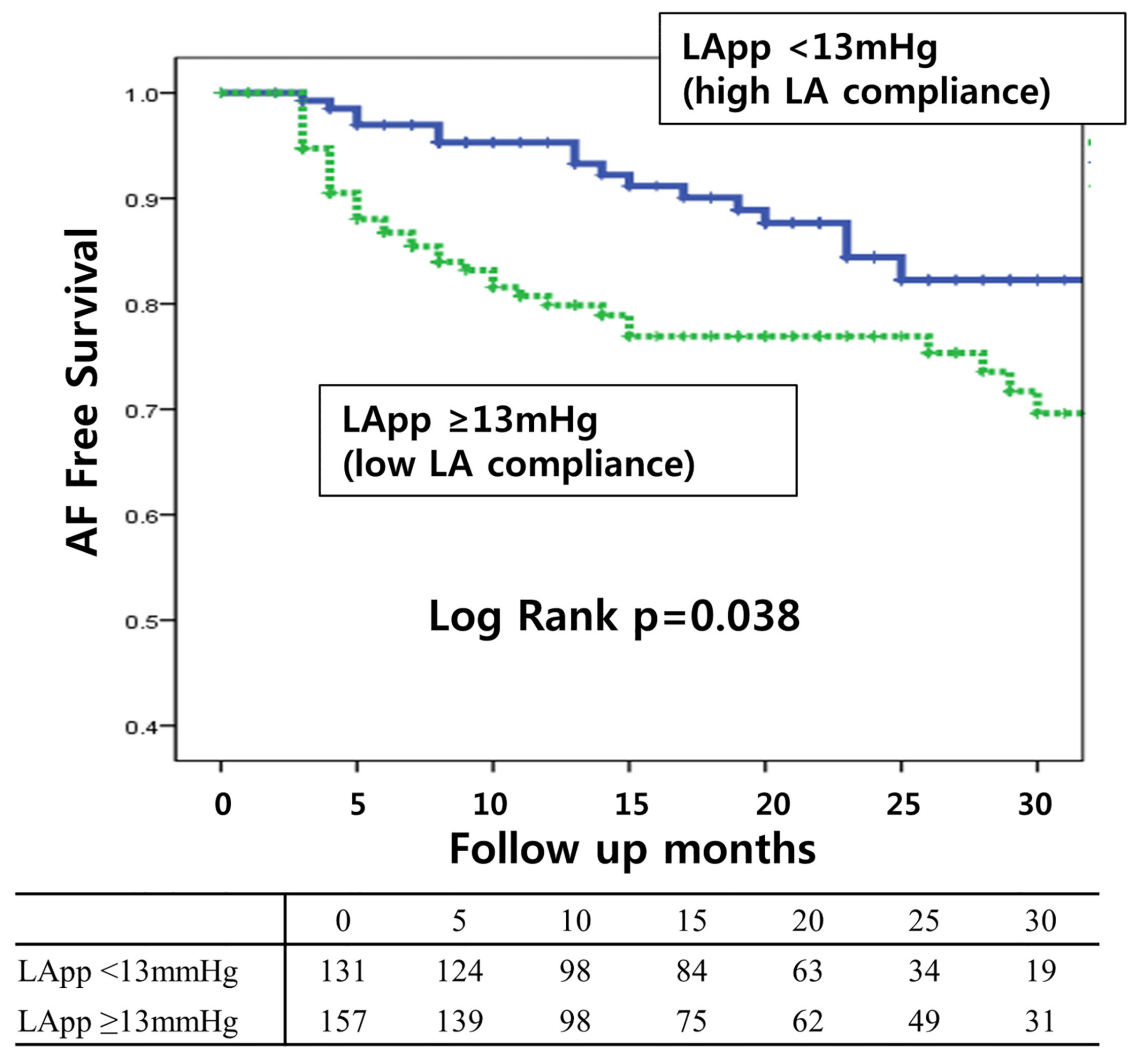
Patients with low LA compliance (LApp≥13mmHg) have a higher recurrence rate than those with high LA compliance.

These results were also consistent in an analysis that included patients with diabetes (S2 Table). Kaplan-Meier analysis for AF-free survival also showed a significantly higher rate of clinical recurrence in patients with high LApp (low LA compliance) than in those with low LApp (high LA compliance; Log Rank: p = 0.038; Fig 3).

## Discussion

In the current study, we explored the clinical significance of reduced LA compliance based on LApp in AF patients with structurally and functionally normal heart to exclude confounding hemodynamic (valvular disease, structural disease, and left ventricular systolic and diastolic dysfunction) [4,12] and metabolic factors (hypertension and diabetes). [13] Low LA compliance was related to a low LA volume index and independently associated with low LA voltage, suggesting LA substrate remodeling without LA enlargement. Low LA compliance was found to be a predictor of clinical recurrence after catheter ablation for AF in structurally and functionally normal heart AF independent of age, gender, and AF type.

### Reduced LA compliance and LA pulse pressure

Stiff LA syndrome has been defined as dyspnea with associated pulmonary hypertension that occurs in the setting of increased LAPs following cardiac surgery. Extensive AF catheter ablation associated with large atrial scars was also reported to be an independent predictor for stiff LA syndrome, [2] although the incidence was very low (1.4%). [3] However, a clear-cut definition and mechanism for LA stiffness in AF patients with structurally and functionally normal heart without cardiac intervention have not yet been clearly determined, despite not being as extreme as stiff LA syndrome. In the current study, we explored the mechanisms of LA stiffness and reduced LA compliance in patients with normal heart AF after excluding hemodynamic, metabolic factors and. And we utilized LApp to represent the LA compliance, similar to the use of aortic pulse pressure as an indicator of aortic compliance.[8]In general, LA compliance can be calculated as the ratio of the change in LA volume to the change in LA pressure (LApp); [5,6] however, precise and instantaneous measurements of LA volume change and pressure are challenging. Therefore, we represented and quantified LA compliance using directly measured LApp, assuming minimal change in LA volume based on previous physiologic studies.[5–7]To minimize confounding factors affecting LA volume, we enrolled AF patients with normal LV systolic and diastolic function and measured LApp at the beginning of the procedure after an overnight fast. As this study was not targeted for symptomatic stiff LA syndrome, there was no gold standard for a threshold LApp that would suggest a reduced LA compliance. However, our definition for reduced LA compliance based on the median value of LApp was a good representation of the degree of LA remodeling and clinical recurrence of AF.

Actually, we tested echocardiographically measured LA capacitance.[14] Although it has negative correlation with LApp (r = -0.477, p<0.001), it did not predict AF recurrence after catheter ablation (median 3.1 mL/mmHg, Log rank p = 0.395). We also calculated atrial contraction by the analysis of LA ejection force in 123 patients whose echocardiogram was measured during sinus rhythm.[15] We think it may be due to the limitation of LA volume measurement by echocardiogram that is dependent on complex geometry, volume status, and rhythm status. However, the patients with high LApp tended to show low LA ejection force (8.6±5.3 vs. 10.5±6.4 kdynes, p = 0.086), and low LA ejection force was associated with high recurrence of AF (HR 0.215, 95% CI 0.054–0.854, p = 0.029).

### Clinical implications of low LA compliance

There are individual differences in the degree of LA remodeling among patients with similar AF durations or AF burdens.[10] LA volume and voltage were variable after adjusting for age, sex, AF type, duration, and other clinical factors in this study and previous studies.[10] Recently, we reported the importance of hemodynamic factors in LA remodeling, which are associated with AF.[4,16]High LAP_peak_ is related to greater LA volume, lower LA voltage, and LV diastolic dysfunction in patients who have undergone AF catheter ablation, and it is also associated with a higher rate of clinical recurrence.[4]Although low LA compliance estimated by LApp was associated with low LA voltage and the recurrence of AF after RFCA in the current study, LA volume was smaller in normal heart AF patients with reduced LA compliance, possibly indicating the early stage of atrial myopathy before LA enlargement. Low endocardial voltage has been known to be an indicator of changes in the cardiac matrix or myocardial scars.[17,18] Therefore, LA scarring may result in low LA compliance and poor clinical outcomes of AF catheter ablation in patients without hemodynamic burden or LA enlargement. This finding is consistent with results of the DECAAF (Delayed Enhancement–MRI determinant of successful Catheter Ablation of Atrial Fibrillation) study, which showed poor clinical outcomes of AF ablation in the group with extensive atrial scarring.[19] Extensive RFCA for AF generates atrial scarring,[20] and a long ablation duration is an independent risk factor for poor clinical outcomes in patients with PeAF. [21] Therefore, there is a need to search for effective antiarrhythmic therapy that avoids extensive tissue scarring caused by aggressive ablation.

### Limitations

This study was an observational cohort study conducted on patients in a registry that included a highly selective group of patients referred for AF catheter ablation. The change in LA volume was not measured to estimate LA compliance, as mentioned above. Instead, we quantified LA compliance by directly measuring LApp and assumed a minimal change in LA volume based on previous physiologic studies. [5–7] Although we excluded other structural and hemodynamic factors as much as possible, we did not exclude sleep apnea, and there was no age limitation (mean age: 58 [18–85] years old). Low LA compliance can be either a perpetuating factor or a result of AF, and further study with atrial tissue characterization by cardiac MRI might be valuable. We measured LAP after cardioversion if the initial rhythm was AF (23%). LApp may have been affected by atrial stunning, despite waiting at least 3minutes after cardioversion, and there was no significant difference between LApp with and without cardioversion (14.5±7.4 mmHg vs. 13.9±5.6mmHg; p = 0.472).We think it is not possible to exclude potential stunning even in patients with paroxysmal AF who showed initial sinus rhythm at the beginning of the procedure. LApp for predicting LA compliance is an invasive parameter, and noninvasive tests[4,22] should be validated as alternatives for wider clinical application of LA compliance in appropriate patient selection for RFCA. Given that the endocardial voltage was measured by point-by-point contact mapping (350–500 points), the voltage map may not represent a spatially homogenous distribution of endocardial voltage. The 3D voltage map analysis was performed with 2D measurements.

## Conclusions

Reduced LA compliance estimated by high LApp was associated with a low LA volume index and independently associated with low LA voltage, suggesting LA substrate remodeling without LA enlargement. Low LA compliance was an independent predictor of clinical AF recurrence after catheter ablation in AF patients with structurally and functionally normal heart.

## Supporting Information

S1 Table Clinical characteristics of patients (including diabetes) (DOCX)

S2 Table Multivariate Cox regression analysis of clinical recurrence of AF after RFCA (including diabetes) (DOCX)
