# Supplementary material for: Low Left Atrial Compliance Contributes to the Clinical Recurrence of Atrial Fibrillation after Catheter Ablation in Patients with Structurally and Functionally Normal Heart
Source: PLoS One. 2015 Dec 1;10(12):e0143853. doi: 10.1371/journal.pone.0143853 (PMC4666672; doi:10.1371/journal.pone.0143853)
Supplement: S1 Table — (DOCX) [file pone.0143853.s001.docx]

**S1 Table. Clinical characteristics of patients (including diabetes)**

|  | **All** | **Normal heart AF** | **Others** | ***p-*value** |
| --- | --- | --- | --- | --- |
|  | (n=1038) | (n=355) | (n=683) |  |
| **Male (n,%)** | 771 (74.3%) | 278 ( 78.3) | 494 (72.3%) | **<0.001** |
| **Age (years)** | 57.7±11.2 | 53.7±10.4 | 59.7±11.1 | **<0.001** |
| **PAF (n,%)** | 704 (67.8%) | 262 (73.8) | 443 (64.9%) | **<0.001** |
| **BSA (m^2^)** | 1.8±0.2 | 1.8±0.2 | 1.8±0.2 | 0.026 |
| **BMI (kg/m^2^)** | 24.9±3.1 | 24.6±2.7 | 25.0±3.2 | 0.053 |
| **CHA2DS2VASc score** | 1.5±1.4 | 0.6±1.0 | 2.0±1.4 | **<0.001** |
| **CHF (n,%)** | 89 (8.6%) | 0 | 89 (13.0%) | NA |
| **Hypertension (n,%)** | 488 (47.0%) | 0 | 488 (71.4%) | NA |
| **Age≥75 (n,%)** | 50 (7.1%) | 4 | 46 | **<0.001** |
| **Diabetes (n,%)** | 140 (13.5%) | 21 (5.9) | 119 (17.4%) | <0.001 |
| **Stroke/ TIA (n, %)** | 125 (12.0%) | 24 (6.8) | 101 (14.8) | **0.001** |
| **Associated structural heart disease^*^** | 261 (37.1%) | 0 | 261 (38.2%) | NA |
| **Coronary artery disease (n,%)** | 142 (20.2%) | 0 | 142 (20.8%) | NA |
| **Valvular heart disease (n,%)** | 75 (10.7%) | 0 | 75 (11.0%) | NA |
| **HCMP (n,%)** | 20 (2.8%) | 0 | 20 (2.9%) | NA |
| **DCMP (n,%)** | 17 (2.4%) | 0 | 17 (2.5%) | NA |
| **Congenital heart disease (n,%)** | 17 (2.4%) | 0 | 17 (2.5%) | NA |
| **LApp (mmHg)** | 15.3±7.6 | 14.1±6.1 | 16.0±8.3 | **<0.001** |

PAF, paroxysmal atrial fibrillation; BSA, body surface area; BMI, body mass index; DCM, dilated cardiomyopathy; HCMP, hypertrophic cardiomyopathy; TIA, transient ischemic attack; LApp, left atrial pulse pressure;
