# Supplementary material for: Low Left Atrial Compliance Contributes to the Clinical Recurrence of Atrial Fibrillation after Catheter Ablation in Patients with Structurally and Functionally Normal Heart
Source: PLoS One. 2015 Dec 1;10(12):e0143853. doi: 10.1371/journal.pone.0143853 (PMC4666672; doi:10.1371/journal.pone.0143853)
Supplement: S2 Table — (DOCX) [file pone.0143853.s002.docx]

**S2 Table. Multivariate Cox regression analysis of clinical recurrence of AF after RFCA (including diabetes)**

|  | **Univariate analysis** | | | **Multivariate analysis** | | | |
| --- | --- | --- | --- | --- | --- | --- | --- |
|  | **HR** | **95% CI** | ***p*-value** | | **HR** | **95% CI** | ***p*-value** |
| **Age** | 1.009 | 0.985-1.033 | 0.48 | | 0.999 | 0.968-1.029 | 0.924 |
| **Male** | 1.113 | 0.583-2.128 | 0.745 | | 1.54 | 0.604-3.929 | 0.366 |
| **Persistent AF** | 1.464 | 0.866-2.476 | 0.155 | | 2.255 | 0.864-5.886 | 0.097 |
| **BSA (m^2^)** | 0.732 | 0.192-2.793 | 0.648 | |  |  |  |
| **BMI (kg/m^2^)** | 1.006 | 0.920-1.100 | 0.892 | |  |  |  |
| **Diabetes** | 2.09 | 0.997-4.383 | 0.051 | | 2.376 | 0.942-5.995 | 0.067 |
| **LA dimension (Echo)** | 1.048 | 1.005-1.093 | **0.027** | | 1.028 | 0.949-1.114 | 0.499 |
| **LA volume index (Echo)** | 1.026 | 1.004-1.048 | **0.021** | | 1 | 0.957-1.045 | 0.997 |
| **LV ejection fraction** | 0.997 | 0.962-1.032 | 0.848 | |  |  |  |
| **E/Em** | 0.995 | 0.901-1.099 | 0.928 | |  |  |  |
| **LAA emptying velocity** | 0.991 | 0.977-1.006 | 0.259 | |  |  |  |
| **LA volume index (3D-CT)** | 1.004 | 0.991-1.018 | 0.53 | |  |  |  |
| **LAA volume index (3D-CT)** | 1.058 | 0.978-1.145 | 0.159 | |  |  |  |
| **Mean LA voltage** | 0.473 | 0.283-0.790 | **0.004** | | **0.444** | **0.245-0.802** | **0.007** |
| **Mean LAA voltage** | 0.814 | 0.661-1.003 | 0.053 | |  |  |  |
| **Ablation time** | 1 | 1.000-1.000 | **0.02** | | **1** | **1.000-1.001** | **0.023** |
| **Early recurrence** | 2.848 | 1.753-4.625 | **<0.001** | | 1.763 | 0.903-3.443 | 0.097 |
| **LApp≥13mmHg** | 1.813 | 1.078-3.050 | **0.025** | | **2.159** | **1.113-4.191** | **0.023** |

BSA, body surface area; BMI, body mass index; LA, left atrium; LV, left ventricle; LAA, left atrial appendage; LApp, left atrial pulse pressure.
